# Genetic Structure and Genome-Wide Association Analysis of Growth and Reproductive Traits in Fengjing Pigs

**DOI:** 10.3390/ani14172449

**Published:** 2024-08-23

**Authors:** Lei Xing, Xuelin Lu, Wengang Zhang, Qishan Wang, Weijian Zhang

**Affiliations:** 1 Shanghai Animal Disease Control Center, Shanghai 201103, China; xinglei2019@163.com (L.X.); xuelinlu6@126.com (X.L.); geligaori@163.com (W.Z.); 2 Department of Animal Science, College of Animal Sciences, Zhejiang University, Hangzhou 310030, China; wangqishan@zju.edu.cn

**Keywords:** Fengjing pigs, genetic diversity, genome-wide association study, SNP chip

## Abstract

**Simple Summary:**

In this study; the Fengjing pig was taken as the research object to explore the degree of its genetic diversity and deeply explore its excellent traits. Analyzing the population’s genetic structure and mining the important candidate functional genes provide a theoretical basis for the conservation and utilization of Fengjing pig germplasm resources.

**Abstract:**

The Fengjing pig is one of the local pig breed resources in China and has many excellent germplasm characteristics. However, research on its genome is lacking. To explore the degree of genetic diversity of the Fengjing pig and to deeply explore its excellent traits, this study took Fengjing pigs as the research object and used the Beadchip Array Infinium iSelect-96|XT KPS_PorcineBreedingChipV2 for genotyping. We analyzed the genetic diversity, relatedness, inbreeding coefficient, and population structure within the Fengjing pig population. Our findings revealed that the proportion of polymorphic markers (P_N_) was 0.469, and the effective population size was 6.8. The observed and expected heterozygosity were 0.301 and 0.287, respectively. The *G*-matrix results indicated moderate relatedness within the population, with certain individuals exhibiting closer genetic relationships. The NJ evolutionary tree classified Fengjing boars into five family lines. The average inbreeding coefficient based on ROH was 0.318, indicating a high level of inbreeding. GWAS identified twenty SNPs significantly associated with growth traits (WW, 2W, and 4W) and reproductive traits (TNB and AWB). Notably, WNT8B, RAD21, and HAO1 emerged as candidate genes influencing 2W, 4W, and TNB, respectively. Genes such as WNT8B were verified by querying the PigBiobank database. In conclusion, this study provides a foundational reference for the conservation and utilization of Fengjing pig germplasm resources and offers insights for future molecular breeding efforts in Fengjing pigs.

## 1. Introduction

Fengjing pigs are renowned for their exceptional reproductive performance, disease resistance, and ability to thrive on roughage [1]. The quality of Fengjing pork is characterized by its fat but non-greasy texture, delicacy, and tenderness, with the renowned “Fengjing Dingti” having won a gold medal at the Panama-Pacific International Exposition [2]. Despite their genetic advantages in reproduction and meat quality, the introduction of foreign pig breeds and industrial development has relegated Fengjing pigs primarily to breeding farms [2]. To fully exploit the advantageous genes of Fengjing pigs, breeding new varieties from these indigenous pigs can promote scientific breeding preservation and enhance the development of high-quality pork products.

With the continuous development of biotechnology, many molecular techniques, such as mitochondrial DNA markers [3], microsatellite markers [4,5], simplified genome sequencing [6], and whole-genome resequencing [7], have been widely used in the evaluation of biological genetic diversity and the identification of new varieties. Single-nucleotide polymorphisms (SNPs), as third-generation molecular markers, exhibit high genetic stability, abundant polymorphic sites, and ease of detection. SNP microarrays, composed of millions of DNA markers fixed on a special silicon chip [8], have been extensively utilized in researching livestock and poultry germplasm characteristics, genetic diversity, and new breed identification. Recent studies have examined the genetic status of indigenous pig breeds, such as Licha Black pigs [9], Liangshan pigs [10], Laiwu pigs [11], Ningxiang pigs [12], and Jiangquhai pigs [13]. The development and application of genome-wide association studies (GWAS) have enabled the screening of SNPs and candidate genes associated with complex traits, including reproductive traits [14,15,16,17,18], meat quality traits [19,20,21,22,23,24,25], carcass traits [26,27], coat color [28,29], and vertebral bone counts [30,31]. These SNPs and candidate genes provide a large number of molecular markers for pigs.

This study aimed to investigate the genetic diversity and structure of the Fengjing pig conservation population based on molecular markers and mine potential candidate genes related to the species’ economically significant traits, such as growth and reproduction. Our results provide a theoretical basis for a certain deeper understanding of Fengjing pig population conservation and pave the way for their exploitation and utilization.

## 2. Materials and Methods

### 2.1. Animals and Phenotype

This study obtained 11 boars and 92 sows from the Shanghai Fengjing Pigs Breeding Farm. All pigs were raised under the same feeding environment and feeding management conditions. The pigs’ birth dates ranged from February 2016 to November 2020 (20 levels). Ear tissues were collected using ear scissors and placed in a centrifuge tube containing anhydrous ethanol. The phenotypic data of the Fengjing pigs were collected, and the descriptive statistics are shown in Table 1. The variation ranges of WW, 2W, 4W, TNB, and AWB were 5.5–6.5, 11–17, 19–26, 5–15.2, and 0.95–1.33, and their average values were 5.99 ± 0.26, 12.72 ± 0.85, 20.64 ± 1.12, 11.79 ± 1.90, and 1.10 ± 0.08, respectively.

### 2.2. Genotype Data

DNA was extracted using the magnetic bead method. Firstly, the tissues were clipped from the original sample tubes with alcohol-sterilized tweezers. The surface protection liquid was blotted with tissue paper. The rice-sized animal tissues were cut with sterilized scissors and put into lysis tubes with 20 μL of ProteinaseK and 300 μL of BufferWL, vortexed and mixed well, then put the tubes into the 56 °C thermostatic bath to lysis for 40 min. Put the completed lysis tubes into the centrifuge for instantaneous centrifugation, transfer the supernatant into 96-deep well plates, and then transfer the supernatant into 96-deep well plates. The samples were lysed in a water bath at 56 °C for 40 min, and then, the lysate tube was put into a centrifuge for instant centrifugation. The supernatant was transferred to a 96-well plate, and the relevant reagents were added. Then, the magnetic beads were adsorbed with a magnetic bar sleeve. The eluted products were, finally, transferred to a 1.5 mL collection tube and stored at a low temperature. The extracted DNA was analyzed by UV spectrophotometer (NanoDrop2000) and gel electrophoresis for quality control, and the qualified DNA samples were used for SNP genotyping with the Beadchip Array Infinium iSelect-96|XT KPS_PorcineBreedingChipV2. SNP genotype data were quality controlled using PLINK v1.90 [32], and only SNPs with autosomal detection rates ≥ 90%, a minimum allele frequency (MAF) > 0.01, and Hardy–Weinberg equilibrium test *p*-values ≥ 0.000001 were used.

### 2.3. Population Genetic Diversity and Structure Analysis

The data after quality control were analyzed using PLINK v1.90. The effective population size (Ne), expected heterozygosity (He), observed heterozygosity (Ho), and proportion of polymorphic markers (P_N_) were calculated for Fengjing pigs, respectively [9]. The kinship coefficients between individuals were calculated using GCTA (V1.94) [33] software, a *G*-matrix was constructed, and a heat map was constructed using R script. The kinship coefficients were calculated as follows:

$$G=\frac{ZZ\prime }{2\sum p_{i}(1-p_{i})}$$

*p_i_* is the frequency of the *i*th allele.

Cluster analysis was carried out using Mega X (V10.0) [34] software using the Neighbor-Joining method (NJ) and clustering the samples based on identical by state (IBS) genetic distances. Finally, combining the results of the genomic kinship analysis and cluster analysis, the boar lineage division was performed to analyze the population structure using the kinship coefficient of ≥0.1 as the criterion.

Firstly, the length of runs of homozygosity (*ROH*) was calculated using PLINK v1.90 for each sample, and the length and number of *ROH* were counted for each Fengjing pig. Then, the inbreeding coefficients based on the *ROH* were obtained by calculating the ratio of the total length of *ROH* fragments to the total length of the autosomal genome of an individual with the following formula:

$$F_{ROH}=\frac{\sum_{k}Length({ROH}_{k})}{L}$$

*k* is the number of *ROH* in an individual, and *L* is the length of the autosomal genome covered by the analyzed data chip.

A population’s average inbreeding coefficient value was obtained by summing the inbreeding coefficient values of all individuals and dividing by the total number of individuals. The formula is as follows:

$$\bar{F_{ROH}}=\sum_{i=1}^{N}\frac{F_{ROHi}}{N}$$

*F_ROHi_* is the inbreeding coefficient value for the individual, and *N* is the total number of individuals.

### 2.4. Genome-Wide Association Study

After a serious filtration step, 23,073 SNPs were retained. Beagle (V5.0) [35] software was used to fill in the missing genotype data, which were then used for the GWAS analysis. The populated genotype data were subjected to principal component analysis using PLINK v1.90 to construct a genomic affinity matrix. Heat maps were drawn using the ggplot2 package of R software, and scatter plots were drawn for the top three principal components with the highest explained variance. This GWAS analyzed growth traits using a general mixed linear model and reproductive traits using a repetitive force mixed linear model. Yu et al. [36] first published the mixed linear model in *Nature Genetics*, and it is widely recognized as the best model for GWAS analysis because it can correct for population structure and complex kinship relationships within a population.

The growth trait analysis model is as follows:

$$y=X\beta +Z_{k}\gamma_{k}+\xi +e$$

y is the phenotype vector, Xβ is the population structure effect (the first 3 principal components of the PCA analysis) and sex and litter effects, $Z_{k}\gamma_{k}$ is the marker effect to be tested, $\xi \sim N(0,K\phi^{2})$ is the polygenic effect, and $e\sim N(0,I\sigma^{2})$ is the residual effect. k in the polygenic effect is the marker-inferred kinship matrix.

The model for analyzing reproductive traits is as follows:

$$y=X\beta +Z_{k}\gamma_{k}+\xi +p+e$$

y is the phenotype vector, Xβ is the population structure effect (the first 3 principal components of the PCA analysis) and the field–year–season effect, $Z_{k}\gamma_{k}$ is the marker effect to be tested, $\xi \sim N(0,K\phi^{2})$ is the polygenic effect, $p\sim N(0,I\sigma_{p}^{2})$ is the systematic environmental effect, and $e\sim N(0,I\sigma^{2})$ is the residual effect. K in the polygenic effect is the marker-inferred kinship matrix.

We used GEMMA [37] and GMAT [38] to solve the mixed linear models. Manhattan and Q–Q plots of the GWAS results were plotted using the qqman package of R software (Version: 0.1.9).

### 2.5. Candidate Gene Annotation

The reference genome information for the corresponding pig was downloaded from the Ensembl website, and significant SNPs were annotated to the corresponding genes using ANNOVAR software (2019) (http://annovar.openbioinformatics.org/en/latest/user-guide/download/, accessed on 19 August 2024) [39]. The annotated candidate genes were then analyzed for GO and KEGG enrichment using the clusterProfiler package [40,41].

## 3. Results

### 3.1. Genetic Diversity of Fengjing Pigs

The Ne of the Fengjing pig conservation farm was 6.8, and the ratio of P_N_ was 0.469. The Ho (0.301) was higher than He (0.287).

### 3.2. Genetic Relationships between and Population Structure of Fengjing Pigs

**Figure 1:**
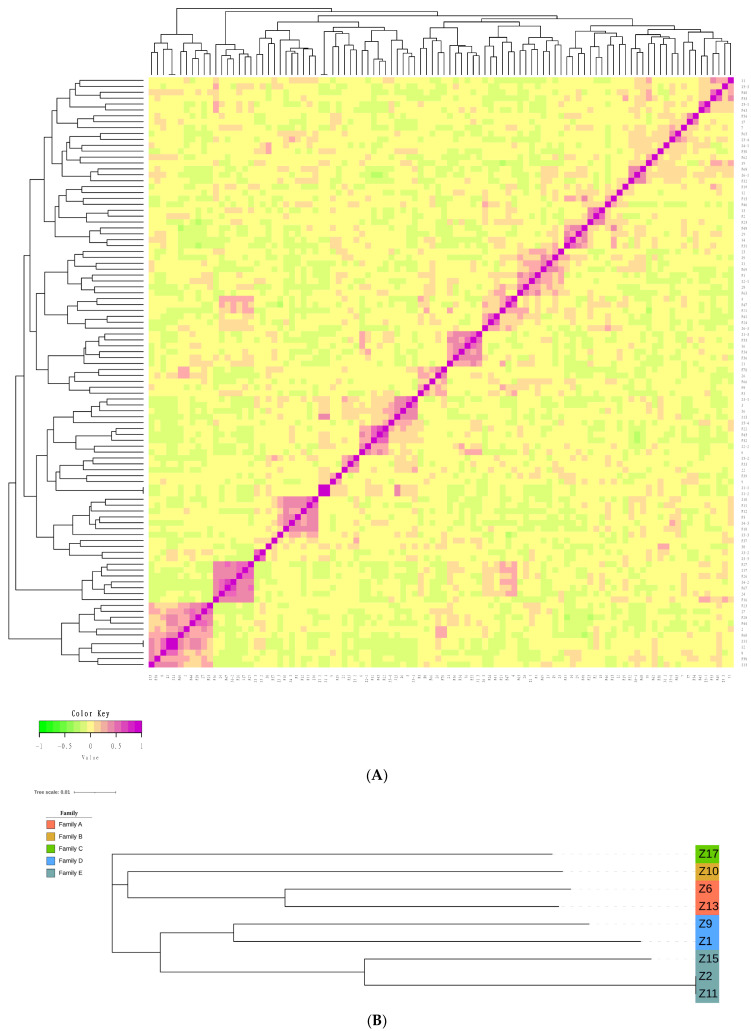

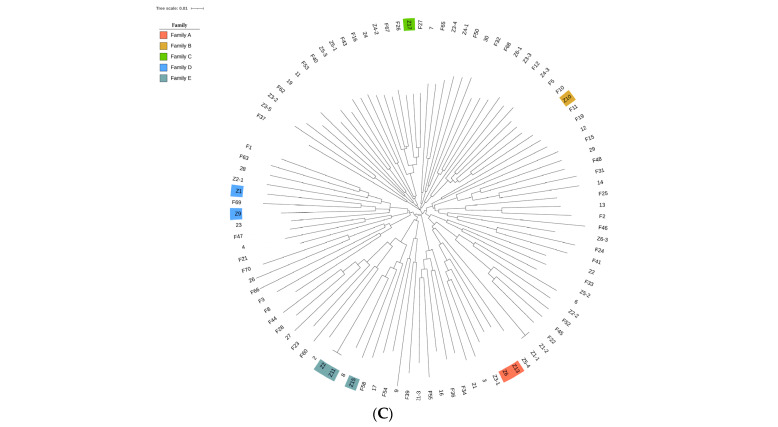
(**A**) *G*-matrix heat map of Fengjing pigs in the conserved population. Each tiny square exhibits the kinship value between different individuals. The closer the color of the squares is to red, the closer the kinship between individuals. (**B**) The phylogenetic tree of Fengjing boars. The numbers are boar ear numbers, and samples marked with the same color in the evolutionary tree diagram were assessed to be of the same lineage. (**C**) Phylogenetic tree of all individuals in this population. Individuals with the same color belong to the same familial lineage.

In this conserved population, the genetic relationship coefficients ranged from −0.2278 to 0.9989 (Supplementary Table S1), and the visualization results of the *G*-matrix are exhibited in Figure 1A. Further analysis of the *G*-matrix of the genomic relationships using SNP loci showed that most Fengjing pigs were moderately related, and some individuals were more closely related. Considering the importance of boars in the breeding process, we analyzed the phylogenetic tree of boars using NJ. As shown in Figure 1B, the phylogenetic tree classified nine boars into five different family lines with similar genetic ancestry. In addition, these individuals were divided into five large families based on the genomic relationships between boars and sows (Figure 1C).

### 3.3. Inbreeding Coefficient Results

**Figure 2:**
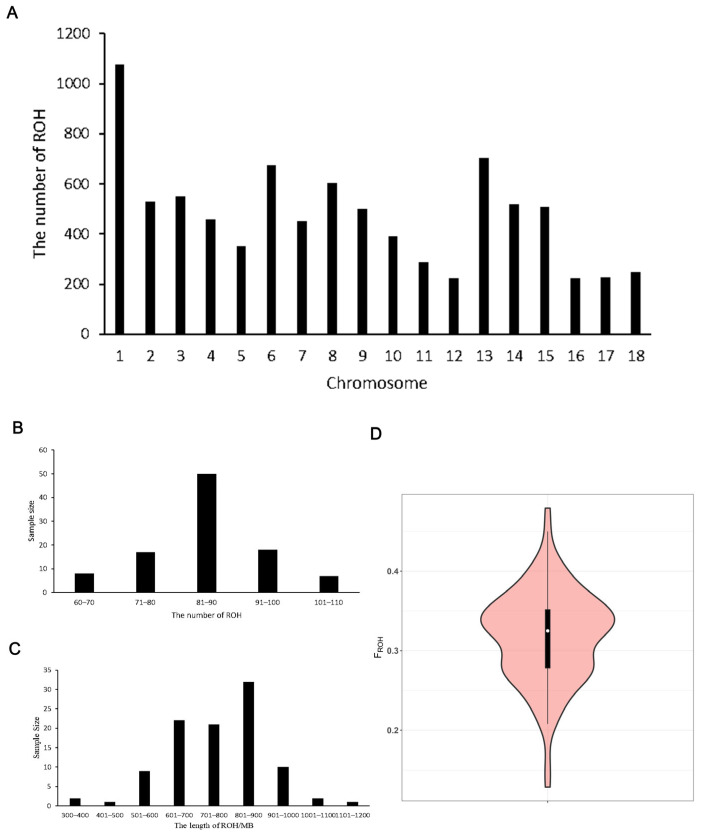
(**A**), Distribution of ROH numbers on the chromosomes in Fengjing pigs. (**B**) Distribution of ROH numbers in Fengjing pigs. (**C**) Distribution of ROH length in Fengjing pigs. (**D**) Distribution of the inbreeding coefficient based on runs of homozygosity in Fengjing pigs.

The results of the genome ROH analysis showed that 8534 ROH fragments were detected in Fengjing pigs, with the highest number of 1078 ROH fragments on chromosome 1 and the lowest number of 222 ROH fragments on chromosome 12 (Figure 2A). The number of ROHs in each Fengjing pig ranged from 62 to 109, averaging 85. The number of individuals with 81–90 ROHs was the highest (Figure 2B). The total length of ROH in Fengjing pigs was 308–1147 Mb, with an average of 762 Mb, and the largest number of individuals had a total length of 801–900 Mb (32 individuals), accounting for 32% of the total length of ROH (Figure 2C). The average inbreeding coefficient of the population was calculated to be 0.318 by counting the ROH of each individual in the population and obtaining the inbreeding coefficient of each individual based on the ROH (Figure 2D).

### 3.4. The Significant SNPs and Genes for Growth Traits

**Figure 3:**
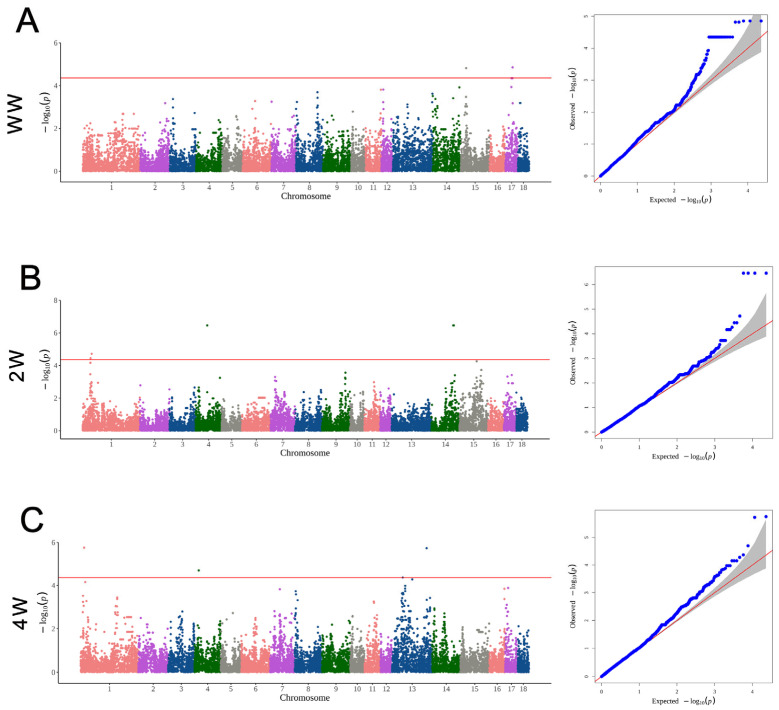
The Manhattan plots and quantile–quantile plots of the GWAS results of the growth traits. (**A**) The Manhattan plots and quantile–quantile plots of the GWAS results of the WW trait. (**B**) The Manhattan plots and quantile–quantile plots of the GWAS results of the 2W trait. (**C**) The Manhattan plots and quantile–quantile plots of the GWAS results of the 4W trait. The red line in the Manhattan plots represents the level of significance. The red line in the quantile–quantile plots is the middle line assuming that the expected value equals the observed value.

The GWAS results are displayed in Table 2 and Figure 3. In detail, five SNPs (CNC10170722, CNC10170723, CNC10170724, CNC10150572, and CNC10150582) located on chromosomes 17 and 15, respectively, were detected to be associated with trait WW, and the genes closest to the five SNPs were DEFB119, DEFB116, HM13, BIN1, ENSSSCG00000015724, GYPC, and ENSSSCG00000023800, respectively. Moreover, seven SNPs located on chromosomes 4, 14, and 1 were associated with 2W, including CNC10041249, CNC10142321, CNC10142322, CNC10142394, CNC10011036, CNC10010898, and CNC10010900, which were, respectively, mapped to genes WNT8B, GOT1, PSD, ROS1, ENSSSCG00000027613, and ENSSSCG00000004231. Regarding the 4W trait, four SNPs reached a significant level. Among these four SNPs, CNC10133689 and CNC10131137 on chromosome 13 were mapped to genes ENSSSCG00000012002, LIPI, and ENSSSCG00000027675. On chromosome 1, the TAB2 and UST genes corresponding to the significant SNP (CNC10010417) were significantly associated with 4W. On chromosome 4, the RAD21 gene corresponding to the significant SNP (CNC10040479) was significantly associated with 4W.

### 3.5. Enrichment Analysis for Growth Traits

**Figure 4:**
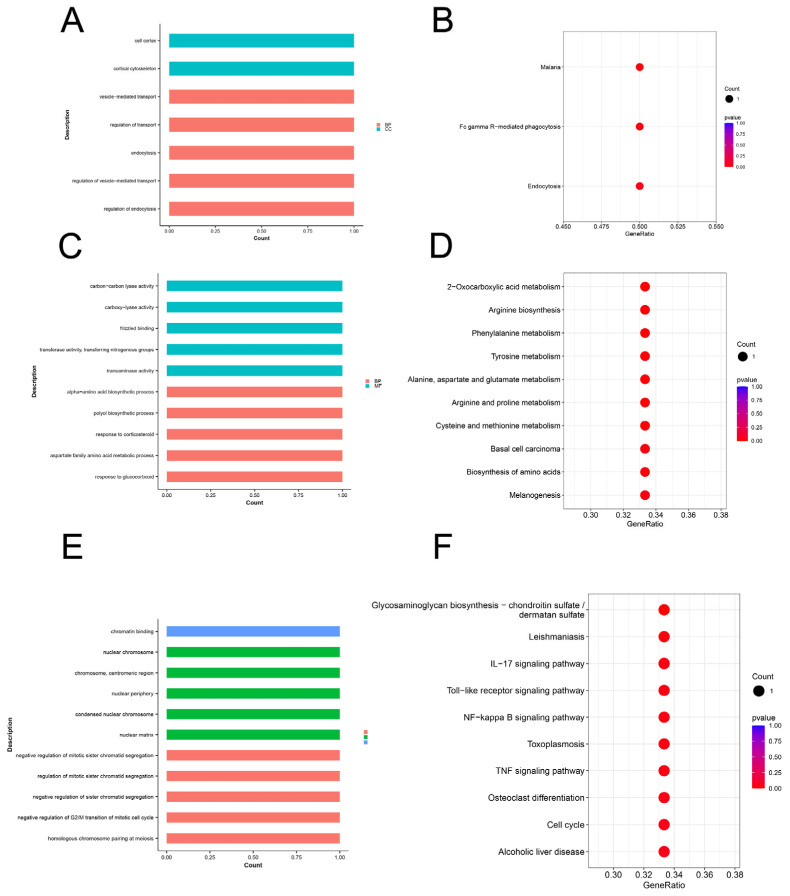
GO annotation and KEGG pathway analysis for candidate genes. (**A**) The GO enrichment analysis of WW candidate genes. (**B**) The KEGG pathways of WW candidate genes. (**C**) The GO enrichment analysis of 2W candidate genes. (**D**) The KEGG pathways of 2W candidate genes. (**E**) The GO enrichment analysis of 4W candidate genes. (**F**) The KEGG pathways of 4W candidate genes.

The corresponding candidate genes were used for the enrichment analysis to obtain more insights into the functions of the 16 related to the indicators of the growth traits (WW, 2W, and 4W). The KEGG and Go enrichment results of the top 10 are shown in Figure 4. The significant GO terms of the WW candidate genes mainly included cortical cytoskeleton, cell cortex, regulation of endocytosis, regulation of vesicle-mediated transport, endocytosis, regulation of transport, and vesicle-mediated transport (Figure 4A). On the other hand, the KEGG pathways of the WW candidate genes are mainly related to Malaria, Fc gamma R-mediated phagocytosis, and Endocytosis (Figure 4B). The significant GO terms of the 2W candidate genes mainly included transaminase activity, transferase activity, transferring nitrogenous groups, frizzled binding, response to glucocorticoid, carboxy-lyase activity, aspartate family amino acid metabolic process, response to corticosteroid, polyol biosynthetic process, carbon–carbon lyase activity, and alpha-amino acid biosynthetic process (Figure 4C). On the other hand, the KEGG pathways of the 2W candidate genes were mainly related to 2-oxocarboxylic acid metabolism, arginine biosynthesis, phenylalanine metabolism, tyrosine metabolism, alanine, aspartate and glutamate metabolism, arginine and proline metabolism, cysteine and methionine metabolism, basal cell carcinoma, biosynthesis of amino acids, and melanogenesis (Figure 4D). The significant GO terms of the 4W candidate genes mainly included homologous chromosome pairing at meiosis, nuclear matrix, condensed nuclear chromosome, nuclear periphery, negative regulation of G2/M transition of mitotic cell cycle, negative regulation of sister chromatid segregation, regulation of mitotic sister chromatid segregation, negative regulation of mitotic sister chromatid segregation, negative regulation of mitotic metaphase/anaphase transition, and negative regulation of cell cycle G2/M phase transition (Figure 4E). On the other hand, the KEGG pathways of the 4W candidate genes were mainly related to glycosaminoglycan biosynthesis–chondroitin sulfate/dermatan sulfate, leishmaniasis, IL-17 signaling pathway, Toll-like receptor signaling pathway, NF-kappa B signaling pathway, toxoplasmosis, tumor necrosis factor (TNF) signaling pathway, osteoclast differentiation, cell cycle, and alcoholic liver disease (Figure 4F).

### 3.6. The Significant SNPs and Genes for Reproduction Traits

**Figure 5:**
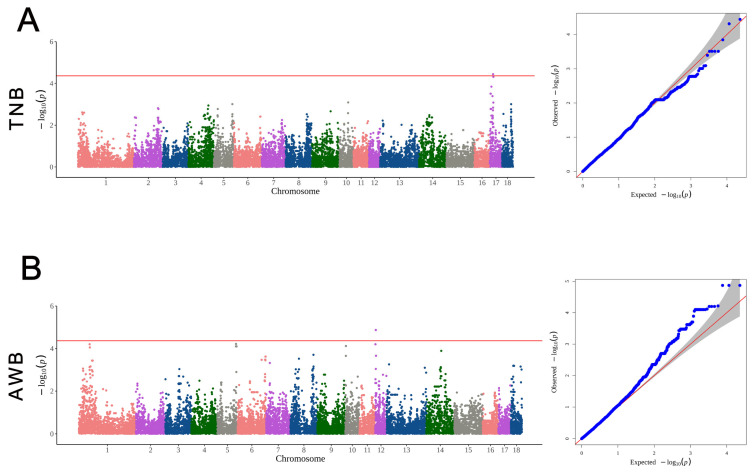
The Manhattan plots and quantile–quantile plots of the GWAS results of the reproduction traits. (**A**) The Manhattan plots and quantile–quantile plots of the GWAS results of the TNB trait. (**B**) The Manhattan plots and quantile–quantile plots of the GWAS results of the AWB trait. The red line in the Manhattan plots represents the level of significance. The red line in the quantile–quantile plots is the middle line assuming that the expected value equals the observed value.

The GWAS results of the reproduction traits are displayed in Table 3 and Figure 5. On chromosome 17, ENSSSCG00000025527 and the HAO1 gene corresponding to the significant SNP (CNC10170350) were significantly associated with TNB. Regarding the AWB trait, three SNPs reached a significant level. Among these three SNPs, CNC10120065, CNC10120066, and CNC10120068 on chromosome 12 were mapped to the genes ENPP7 and TIMP2.

### 3.7. Enrichment Analysis for Reproduction Traits

**Figure 6:**
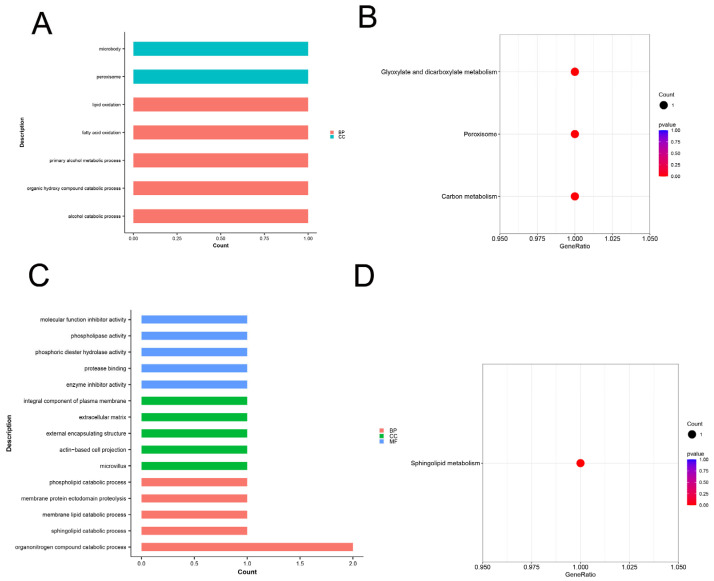
GO annotation and KEGG pathway analysis for candidate genes. (**A**) The GO enrichment analysis of the TNB candidate genes. (**B**) The KEGG pathways of the TNB candidate genes. (**C**) The GO enrichment analysis of the AWB candidate genes. (**D**) The KEGG pathways of the AWB candidate genes.

The corresponding candidate genes were used for the enrichment analysis to obtain more insights into the functions of the four related to the indicators of the reproduction traits (TNB and AWB). The top 10 KEGG and Go enrichment results are shown in Figure 6. The significant GO terms of the TNB candidate genes mainly included alcohol catabolic process, organic hydroxy compound catabolic process, primary alcohol metabolic process, fatty acid oxidation, lipid oxidation, fatty acid catabolic process, monocarboxylic acid catabolic process, peroxisome, microbody, and lipid modification (Figure 6A). On the other hand, the KEGG pathways of the TNB candidate genes were mainly related to glyoxylate and dicarboxylate metabolism, peroxisome, and carbon metabolism (Figure 6B). The significant GO terms of the AWB candidate genes mainly included organonitrogen compound catabolic process, sphingolipid catabolic process, membrane lipid catabolic process, microvillus, membrane protein ectodomain proteolysis, phospholipid catabolic process, enzyme inhibitor activity, membrane protein proteolysis, regulation of DNA replication, and protease binding (Figure 6C). On the other hand, the KEGG pathways of the AWB candidate genes were mainly related to sphingolipid metabolism (Figure 6D).

## 4. Discussion

### 4.1. Analysis of Genetic Structure

The genetic diversity of the Fengjing pig population can be assessed to understand its current conservation status. P_N_, Ne, He, and Ho were the main parameters for evaluating population diversity. The P_N_ of Fengjing pigs was 0.469, which indicated that the population had a low genetic polymorphism compared to other local breeds, such as Tongcheng pigs [41] and Licha Black Pigs [9]. The Ne of Fengjing pigs was 6.8, lower than that of other local pig breeds in China [10,42]. A small effective population size suggests limited migration and interbreeding during evolution, leading to low genetic variation [42]. Ho is the ratio of the number of individuals that are heterozygous at a given site to the total number of individuals in a population, and He is the probability that heterozygosity occurs at any site in any individual in a population [43]. This study found that Ho (0.301) was higher than He (0.287), suggesting that the Fengjing pig population may have been introduced into other varieties, resulting in impurity. The small population size of the Fengjing pig breeding herd and years of closed breeding have led to low genetic diversity and an effective population content, a common issue in Chinese livestock and poultry breeding farms. It is necessary to introduce new lines of Fengjing pigs from outside to expand the effective population content and increase its genetic diversity.

In the process of Fengjing pig conservation, the production data are recorded by handwriting, under which genealogical errors or lack of genealogy often occur, which is not conducive to the conservation work. The kinship matrix constructed based on genomic information can more accurately reflect the relationship between individuals. Visualization of the *G*-matrix shows that most individuals in the Fengjing pig population are distantly related, while a few are more closely related. Loss of ear tags and recording errors in production management will lead to mistakes in traditional genealogical records. The use of whole genome SNP microarray technology to construct a molecular genealogy of breeding pigs is more accurate, which can make up for the shortcomings of traditional genealogy and solve the technical problems of the bloodline and family division of local pig breeding groups. In this study, when we analyzed the breeding herd of Fengjing pigs by SNP microarray technology, we found five breeding boar families, which seriously affected the breeding work and suggested that it was not recommended to breed males and females of the same family line. At the same time, because of the low number of boar pedigrees in the group at present, it is recommended to subsequently select the offspring of mating with individuals distantly related to the sows for breeding retention and gradually reduce the inbreeding coefficient of the group.

The length and frequency of ROH, a continuous fragment of a pure genotype within an individual, results from the complete transmission of a homozygous haplotype from parent to offspring and can reflect the population history. The length and frequency of ROH fragments can reflect the population history. An individual’s inbreeding coefficient can be effectively evaluated using ROH fragments. The average inbreeding coefficient of the Fengjing pig population was calculated to be 0.318, indicating some degree of inbreeding.

### 4.2. Analysis of Candidate Genes for Growth

Nineteen significant genes were annotated for gene function, and two candidate genes were found to be the most likely to affect the growth traits. The DEFB116 and DEFB119 genes are associated with the defense response [44]. HM13 is involved in glycolysis and is one of the essential markers of energy metabolic homeostasis and response to oxidative stress [45]. Masuko Katoh et al. identified the cow WNT8B gene within the NW_001494361.1 genome sequence, suggesting that WNT8B is a potential inducer of hepatocyte differentiation [46]. Analysis of WNT8B gene enrichment in this study indicated that WNT8B plays a role in the biological process of the Wnt signaling pathway (GO:0016055). The Wnt signaling network is of great interest because of its extensive involvement in processes including embryonic skeletal myogenesis and adult skeletal muscle regeneration. The classical Wnt signaling pathway mainly regulates embryonic skeletal muscle formation and satellite cell differentiation during skeletal muscle regeneration. In contrast, the non-classical Wnt signaling pathway plays a vital role in maintaining the skeletal muscle satellite cell pool and promoting myofiber hypertrophy [47,48]. Dysregulation of the Wnt signaling network can lead to impaired embryonic skeletal muscle development or adult skeletal muscle dysfunction [49]. The WNT8B gene may be a candidate gene associated with 2W. In addition to maintaining the chromatin architecture during the normal cell cycle and DNA DSB repair, RAD21 is also linked to various other functions, including apoptosis and hematopoiesis [50]. RAD21 gene enrichment analysis indicated its role in mitotic nuclear division regulation (GO:0007088) and the meiosis I cell cycle process (GO:0061982). The RAD21 gene may be a candidate gene associated with 4W. The WNT8B gene and the RAD21 gene have been verified as related to body weight traits by querying the PigBiobank database [51].

### 4.3. Analysis of Candidate Genes for Reproduction Traits

Four significant genes were annotated for gene function, with HAO1 identified as the most likely candidate affecting reproduction traits. The HAO1 gene was related to fat metabolism [52]. HAO1 gene enrichment analysis indicated that the HAO1 gene plays a role in the fatty acid catabolic process (GO:0009062) and lipid catabolic process (GO:0016042). n-6 PUFA and n-3 PUFA are involved in lipid metabolism, sugar metabolism, amino acid metabolism, and other metabolic activities in animals and have the function of improving mental diseases, obesity, reproduction disorders, and so on, and they are essential fatty acids for animals [53,54,55,56,57,58]. Excessive n-3 PUFA intake induces oxidative stress, increases fat deposition, and inhibits the growth and reproduction of the animal organism, and excessive n-6 PUFA intake increases the production of inflammatory factors and causes fat deposition [59,60]. Studies in mammals and ruminants have shown that PUFA is strongly associated with follicular cell development, oocyte maturation and quality, and early embryo quality [61,62]. The HAO1 gene may be a candidate gene associated with TNB. The HAO1 gene has been verified as related to TNB traits by querying the PigBiobank database [51]. TIMP2 has been shown to be a human obesity-related gene that inhibits the role of metalloproteinases in adipocyte differentiation [63,64].

## 5. Conclusions

The results showed that the population’s genetic diversity was low, the overall kinship was distant, and inbreeding existed among some individuals. There was a high risk of inbreeding among the population, which should be strictly managed to avoid inbreeding in order to promote the conservation and use of genetic resources of the Fengjing pig. We detected 20 SNPs significantly associated with the WW, 2W, 4W, TNB, and AWB traits, identifying WNT8B, RAD21, and HAO1 as candidate genes affecting the 2W, 4W, and TNB, respectively. This study lays the genetic foundation for the subsequent selection and breeding of Fengjing pigs by molecular breeding methods and provides reliable genetic resources for improving the economically important traits in pigs.

## Figures and Tables

**Table 1:** Summary statistics of body measurement and reproduction traits in the Fengjing population.

Trait	N	Mean	SD	Minimum	Maximum	CV (%)
WW	62	5.99	0.26	5.5	6.5	4.35%
2W	102	12.72	0.85	11	17	6.67%
4W	102	20.64	1.12	19	26	5.45%
TNB	90	11.79	1.90	5	15.2	16.16%
AWB	90	1.10	0.08	0.96	1.33	7.00%

WW = weaning weight, 2W = 2-month-old weight, 4W = 4-month-old weight, TNB = total number born, and AWB = average weight born alive.

**Table 2:** Significant SNPs for the growth traits of Fengjing pigs by chip data.

Trait	SSC	SNPs	Position	Allele	MAF	*p*-Value	Candidate Genes	Distance (bp)
WW	17	CNC10170722	39945627	C/A	0.15	1.4 × 10^−5^	DEFB119 DEFB116	13,972 51,035
	17	CNC10170723	39980954	T/C	0.15	1.4 × 10^−5^	DEFB119 DEFB116	49,299 15,708
	17	CNC10170724	40081486	C/T	0.15	1.4 × 10^−5^	HM13	within
	15	CNC10150572	29322127	C/T	0.18	1.52 × 10^−5^	BIN1 ENSSSCG00000015724	264,892 79,072
	15	CNC10150582	29648112	G/A	0.18	1.52 × 10^−5^	GYPC ENSSSCG00000023800	22,639 861,666
2W	4	CNC10041249	63954894	G/A	0.02	3.44 × 10^−7^	WNT8B	652
	14	CNC10142321	120080503	C/T	0.02	3.44 × 10^−7^	GOT1	within
	14	CNC10142322	120080803	G/A	0.02	3.44 × 10^−7^	GOT1	within
	14	CNC10142394	123290079	C/T	0.02	3.44 × 10^−7^	PSD	within
	1	CNC10011036	49820676	T/C	0.22	1.91 × 10^−5^	ROS1	within
	1	CNC10010898	43672115	G/A	0.20	3.57 × 10^−5^	ENSSSCG00000027613 ENSSSCG00000004231	140,659 115,540
	1	CNC10010900	43741515	C/T	0.20	3.57 × 10^−5^	ENSSSCG00000027613 ENSSSCG00000004231	210,059 46,140
4W	1	CNC10010417	18998864	T/C	0.02	1.75 × 10^−6^	TAB2 UST	95,072 296,182
	13	CNC10133689	188376810	C/T	0.26	1.86 × 10^−6^	ENSSSCG00000012002 LIPI	525,216 956,415
	4	CNC10040479	23082238	A/G	0.01	2 × 10^–5^	RAD21	within
	13	CNC10131137	58006732	A/G	0.46	4.27 × 10^–5^	ENSSSCG00000027675	within

Note: MAF is the minimum allele frequency. SSC is an abbreviation for sus scrofa chromosome.

**Table 3:** Significant SNPs for the reproduction traits of Fengjing pigs by chip data.

Trait	SSC	SNP	Position	Allele	MAF	*p*-Value	Candidate Genes	Distance (bp)
TNB	17	CNC10170350	18770198	G/T	0.12	3.61 × 10^−5^	ENSSSCG00000025527 HAO1	1,086,671 44,229
AWB	12	CNC10120065	3097741	T/C	0.14	1.35 × 10^−5^	ENPP7 TIMP2	410,670 196,212
AWB	12	CNC10120066	3106253	G/A	0.14	1.35 × 10^−5^	ENPP7 TIMP2	419,182 187,700
AWB	12	CNC10120068	3119720	C/T	0.14	1.35 × 10^−5^	ENPP7 TIMP2	432,649 174,233

## Data Availability

The dataset used and analyzed during the current study is available from the corresponding author upon reasonable request.

## Supplementary Materials

The following supporting information can be downloaded at https://www.mdpi.com/article/10.3390/ani14172449/s1, Table S1: The kinship coefficient of Fengjing pig population.
